# Reservoir characterization of the Abu Roash D Member through petrography and seismic interpretations in Southern Abu Gharadig Basin, Northern Western Desert, Egypt

**DOI:** 10.1038/s41598-024-58846-6

**Published:** 2024-04-18

**Authors:** Ibrahim Lasheen, Ahmed M. Noureldin, Ahmed Metwally

**Affiliations:** 1 Geophysics Department, Faculty of Science, Cairo University, Giza, 12613 Egypt; 2 The General Petroleum Company, Nasr City, Cairo, 11765 Egypt

**Keywords:** Southern Abu Gharadig Basin, Petrographic analysis, Seismic interpretation, Porosity types, Diagenetic effect, Abu Roash D, Northern western desert, Egypt, Geology, Geophysics, Sedimentology

## Abstract

This research combines petrography and seismic analysis to assess the Upper Cretaceous Abu Roash (AR)/D’s carbonate member composition in the Southwest Abu-Sennan oil field in the Southern Abu Gharadig Basin within the Northern Western Desert of Egypt. Various datasets were used, including petrographic thin sections, and electrical well logs for four stratigraphic wells (01, -02, -03, and, -04), along with a time domain seismic dataset covering the study area. Petrographic analysis across multiple depths and intervals has provided valuable insights. Well-01 demonstrates mud-wackstone with diverse mineral components at 1671–74 m MD, indicating favorable reservoir quality. Well-02 exhibits diverse compositions at intervals 1740–43 m MD and 1746–49 m MD, also showcasing good reservoir quality. Well-03 reveals a packstone rock type at 1662–65 m MD with favorable reservoir characteristics. Well-04 displays peloids Wack-Packstone and Oolitic Packstone at intervals 1764–67 m MD and 1770–73 m MD, respectively, both indicating good reservoir quality. Integrating the petrography and seismic attributes results concerning the structural level of AR/D concerning the used wells, it's evident that Well-03 stands out due to its relatively high structural level, drilled near a major fault, revealing distinct fracture sets that contribute to a notably high reservoir quality as depicted in the RMS amplitude and Ant track attributes maps. AR/D reservoir levels in wells 02, and, 04, are relatively positioned at structurally lower levels, and face challenges with overburden pressure and mechanical compaction, resulting in diminished facies quality for the reservoir. Seismic attributes like the Ant track and RMS amplitude indicated that the presence of fractures within the AR/D Member's carbonate is linked to the prevalence of interpreted normal faults. The implemented procedure in this research can be applied to enhance comprehension of AR/D carbonate reservoirs in adjacent regions, thereby increasing the hydrocarbon exploration possibilities.

## Introduction

Petrography is an essential tool in reservoir characterization in detailed geological studies and provides crucial information about rocks’ mineralogical composition, texture, and diagenetic history^1^. This information is essential for interpreting reservoir quality, pore types, and fluid distribution. On the other hand, seismic interpretation offers a view of subsurface structures through seismic data analysis^2–5^. This study integrates the available seismic data with petrography enhances the delineation of structural and stratigraphic features, providing a more accurate depiction of reservoir geometry and architecture^6^. There are some limitations in data availability and quality, affecting the study's overall precision.

**Figure 1:**
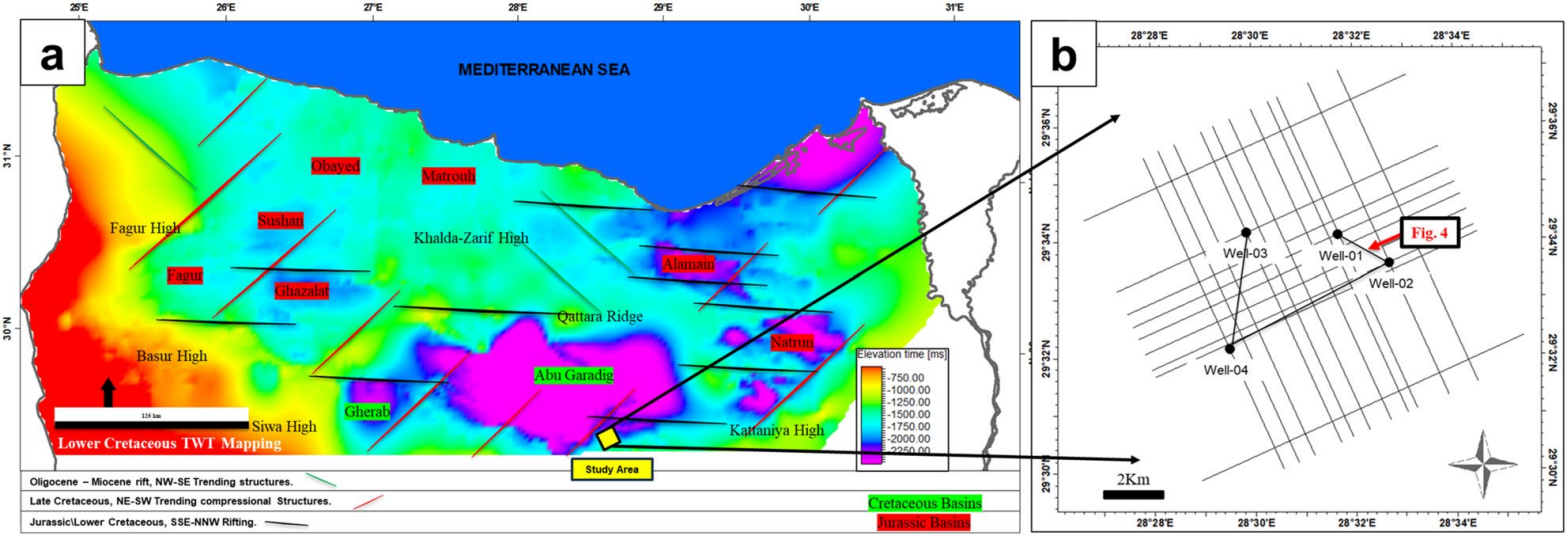
(**a**) Regional Two Way Time (TWT) near the lower Cretaceous level across the Northern Egyptian Western Desert (Updated after^11–13^) (**b**) location for the Southern Abu Gharadig area and the data used in this study.

This study aims to assess the composition and reservoir quality of the Upper Cretaceous member Abu Roash AR/D’s carbonate member in the SWS oil field in the Southern Abu Gharadig Basin within the Northern Western Desert of Egypt to address the key deficiencies in the study area^7–10^. The study area includes wells drilled away from the intended structure, leading to the identification of unfavorable facies. Additionally, it lacks research focusing on carbonate reservoirs. The study area is the Southwest Abu-Sennan (SWS) oil field in the Southern Abu Gharadig Basin within the Northern Western Desert of Egypt lies between Latitudes 29°32′ to 29°35′ North and longitudes 28°30′ to 28°35′ East (Fig. 1).

## Geologic settings

### Stratigraphy

**Figure 2:**
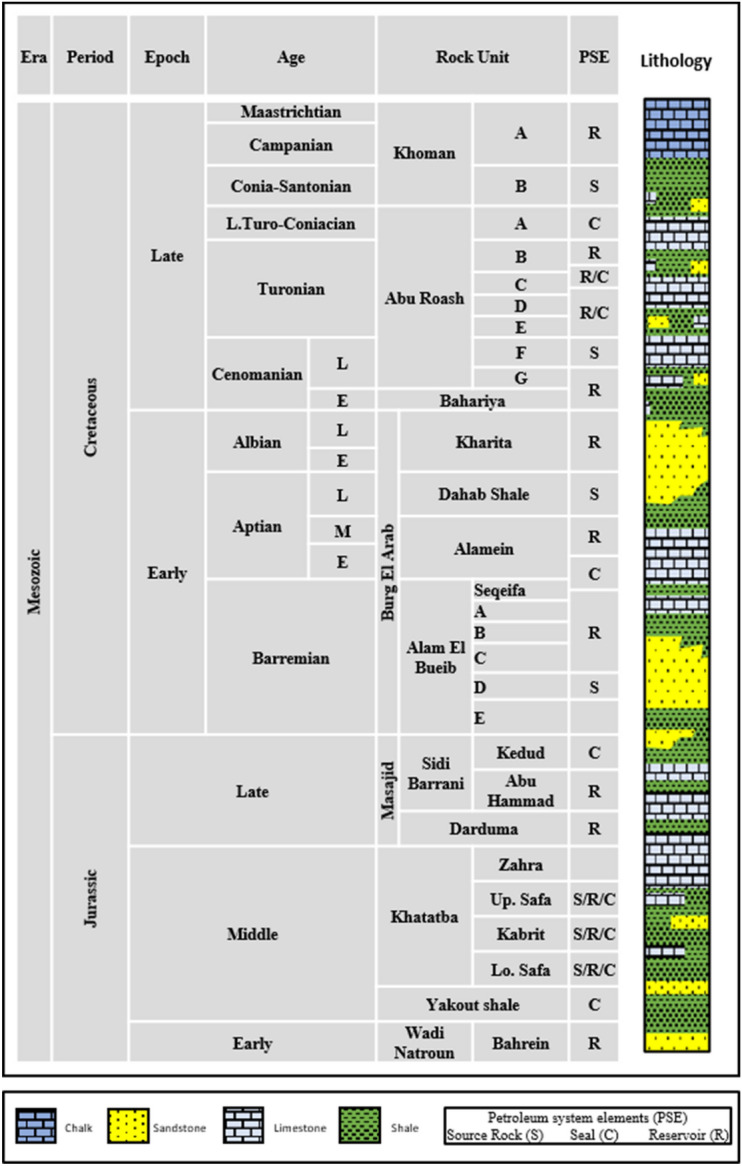
Lithostratigraphic geological rock units of the Northern-Western Desert, Egypt^7–10, 15,16^.

The petroleum systems in the Northern Egyptian Western Desert have been extensively documented by^7–10,12^. These systems, characterized by abundant organic matter source rocks within extended rift basins, have been highlighted by^7–10,14,15^. Figure 2 presents a comprehensive stratigraphical column of the Northern Egyptian Western Desert.

The AR/D carbonate reservoir in the Southern Abu Gharadig Basin, situated in the northern Egyptian Western Desert, is a significant hydrocarbon-bearing formation within the Upper Cretaceous strata^17^, specifically in the Abu Roash Formation. This reservoir plays a crucial role in the region's overall hydrocarbon potential.

### Paleoenvironmental conditions

During the Upper Cretaceous, the northern Egyptian Western Desert underwent a complex interplay of marine and non-marine conditions^18^. The AR/D reservoir likely originated in a shallow marine setting, influenced by periodic sea-level fluctuations. The Abu Roash Formation, known for its Upper Cretaceous carbonate-rich sequences, exhibits alternating layers of limestone, dolomite, and shale, reflecting diverse depositional environments. In the Southern Abu Gharadig Basin, a thick limestone body intercalated with thin shale streaks, exhibiting various colors and textures, is reported in^19^. Macrofaunal and microfaunal content in the AR/District indicates shallow marine carbonates with detrital clastic material influx.

### Source rock and migration

The regional hydrocarbon source rock in the Southern Abu Gharadig Basin is associated with the Jurassic and Upper Cretaceous aged formations. Organic-rich shales within these formations served as prolific source rocks, generating hydrocarbons that migrated upward and accumulated in the porous and permeable intervals of the possible reservoirs. Diagenetic processes, including cementation, dolomitization, and fracturing, significantly influenced the reservoir quality of the AR/D carbonate interval. Understanding the diagenetic history is crucial for reservoir characterization and production optimization.

### Structural framework

The geological history and successive structural events in the Northern Egyptian Western Desert, particularly in the Abu-Gharadig Basin, are characterized by EW and ENE-WSW trending faults spanning the Tertiary, Cretaceous, and Jurassic periods^7–10,20^. The Abu-Gharadig Basin exhibits folds, rotated fault blocks, faults, and unconformities, with their dominance outlined by^11^. These geological structures play a crucial role in the overall architecture of the basin^21^. The structural framework of the Southern Abu Gharadig Basin mirrors that of the Northern Egyptian Western Desert and Abu-Gharadig Basin, characterized by northwest-southeast trending anticlines and synclines dissected by EW and ENE-WSW trending faults. These structural features indicate tectonic activity during the Cretaceous^22,23^ and have played a key role in the trapping and accumulation of hydrocarbons in the AR/D reservoir.

In conclusion, the AR/D carbonate reservoir is a complex geological entity shaped by stratigraphic, structural, and diagenetic factors. Ongoing research and exploration activities refine our understanding of the reservoir, facilitating the sustainable development of hydrocarbon resources in the northern Egyptian Western Desert.

## Material and methods

The study incorporated data from four stratigraphic-control wells (01, 02, 03, and 04) and time-domain seismic data covering the SWS hydrocarbon field characterized by being zero phase with frequency ranges from 10 to 45 Hz (Fig. 1), mapping was conducted using (Petrel E&P software, version 2017, https://www.software.slb.com/products/petrel)^24^ was used during the study. Supplementary data were gathered and examined, including well-head surface locations, formations tops, and Check-Shot surveys. Petrophysical parameters data, encompassing Gamma ray log (GR), Micro Spherical focused log (MSFL), Shallow Latero Log (LLS), Deep Latero Log (LLD), Density (RHOB), and Neutron (NPHI), were incorporated.

Petrography analysis and the assessment of visual porosity were conducted on thin sections related to four wells presented in^19^ employing^25^ rock classification. The analysis facilitated the determination of reservoir components, porosity types, and diagenesis processes such as cementation, recrystallization, and compaction.

**Figure 3:**
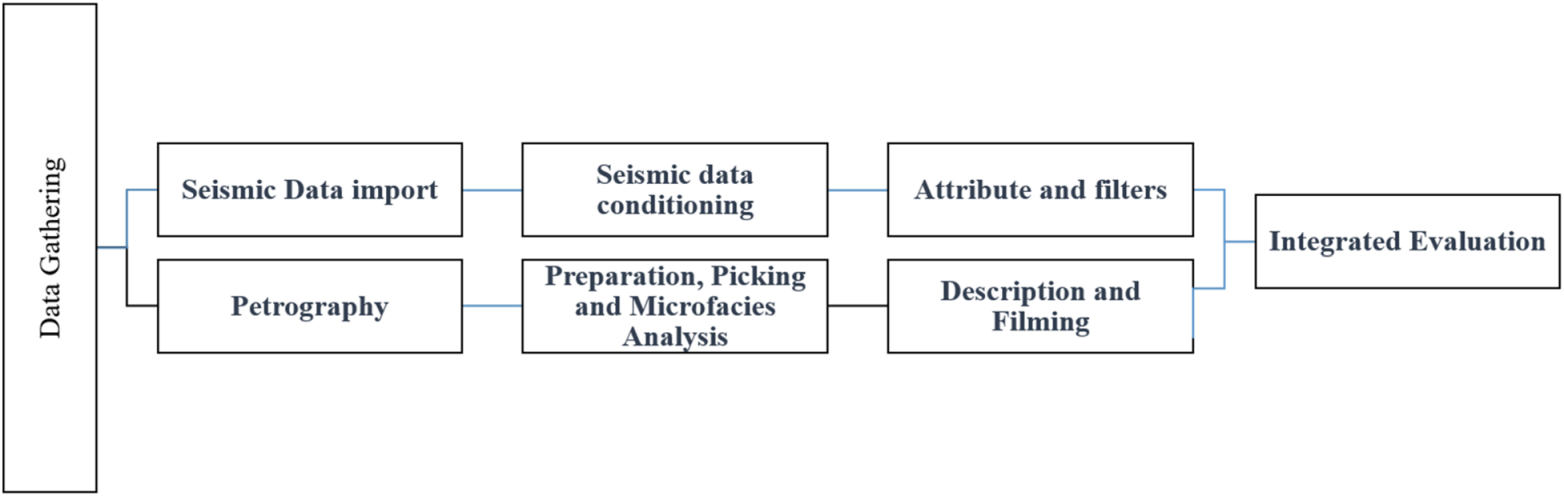
The workflow defines the practical steps followed in the structural analysis.

**Figure 4:**
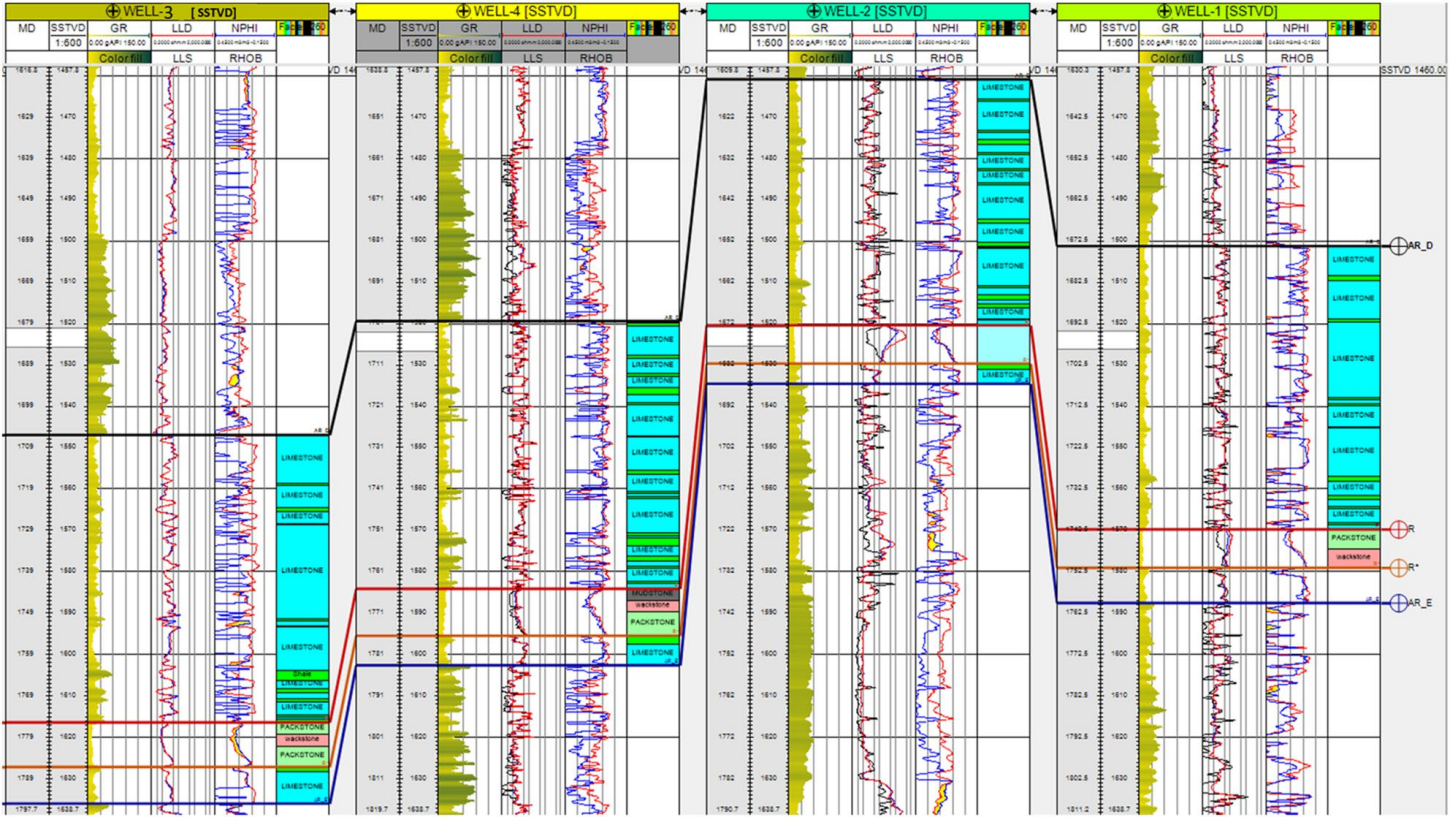
Stratigraphic correlation panel passing by Well-01, Well-02, Well-04, and Well-03 showing the distribution of AR/D member.

The workflow defines the practical steps followed in the study is represented in Fig. 3. A stratigraphic correlation was performed between the wells concerning the Upper Cretaceous members, as depicted in Fig. 4. A lithostratigraphic analysis was also performed based on ditch cuttings investigation and mud log description across multiple wells in the designated area. Seismic attributes^26,27^ such as the Ant track and RMS amplitude to recognize edge geometries and stratigraphical anomalies, as outlined by^28,29^ were performed.

## Results

### Petrography analysis of the AR/D member in different wells

Our investigation into the AR/D Member in various wells within the Southern Abu Gharadig Basin is fundamental to interpreting the geological complexities of this carbonate reservoir. The analysis of 01, 02, 03, and 04 wells has yielded comprehensive insights into the lithological variations and reservoir quality of the AR/D Member, significantly enhancing our understanding of its hydrocarbon potential.

In the dominion of petrographic analysis, the assessment of visual porosity^30^ from thin sections plays a pivotal role in unraveling the geological intricacies of subsurface formations. Integrating this microscopic examination with porosity charts provides a comprehensive understanding of the reservoir characteristics. The porosity chart, graphing depth on the Y-axis against porosity percentage on the X-axis, becomes a visual representation of the evolving porosity profile throughout the AR/D reservoir intervals. As the chart unfolds, distinct patterns emerge, showcasing the intricate dance of different types of porosities—secondary interparticle porosity (SWP), Moldic porosity (MO), and fracture-related porosity (FR), contributing to the overall reservoir heterogeneity and influencing fluid flow dynamics in subsurface environments. Thus, the combination of visual porosity assessments from thin sections and the graphical representation in porosity charts becomes a powerful tool for geoscientists and petroleum engineers in deciphering the hidden complexities beneath the Earth’s surface.

The examination of visual porosity from thin sections holds paramount significance in geological and petrological studies, providing invaluable insights into the physical characteristics of rocks. Thin sections, prepared by slicing rock samples into ultra-thin slices, allow for detailed microscopic analysis of mineral constituents and their spatial arrangements. Visual porosity analysis aids in the identification and quantification of pore spaces within rocks, contributing to a comprehensive understanding of reservoir properties, fluid flow dynamics, and the overall geologic history of a region. Recent advancements in imaging techniques and analytical tools have enhanced the precision and efficiency of visual porosity assessments, facilitating more accurate interpretations of subsurface processes. A notable recent reference in this field is the work of^31^ where innovative methodologies were employed to investigate porosity variations in sedimentary rocks, emphasizing the continued evolution and refinement of porosity analysis techniques.

### AR/D member petrography analysis in Well-01

#### The depth interval of 1671-74 meters

The composition of the sedimentary profile indicates a prevalence of mud-wackstone. This type of rock is characterized by a minor presence of benthic foraminifera and very minimal terrigenous clays. The plate description also notes rare occurrences of Echinoides, ostracods, detrital quartz, dolomite, ferroan calcite, pelecypodes, ferroan dolomite, pyrite, phosphatic fragments, and glaucony. Despite the scarcity of these elements, the reservoir quality at this depth is notably good. The presence of minerals and fossils in the mud-wackstone suggests a sedimentary environment conducive to their preservation, contributing to the overall quality of the reservoir.

#### The depth interval of 1674–77 meters

The sedimentary profile transitions to a wackstone rock type. This lithological unit primarily consists of a minor proportion of benthic foraminifera and terrigenous clays, with very minor occurrences of pelloides and dolomite within the rock matrix. Despite the varied composition, the reservoir quality is assessed as moderate, with foraminifera and clays playing a significant role in the overall rock character.

#### The depth interval of 1677-1680 meters

The subsurface strata are characterized as wackstone, primarily consisting of minor terrigenous clays, with lesser amounts of dolomite and benthic foraminifera. Sparse occurrences of Echinoides, ostracods, pelloides, pyrite, detrital quartz, and ferroan dolomite contribute to the overall composition. The reservoir quality at this depth interval is deemed moderate, indicating a balance between porosity and permeability within the rock structure.

#### The depth interval of 1680-1683 meters

**Figure 5:**
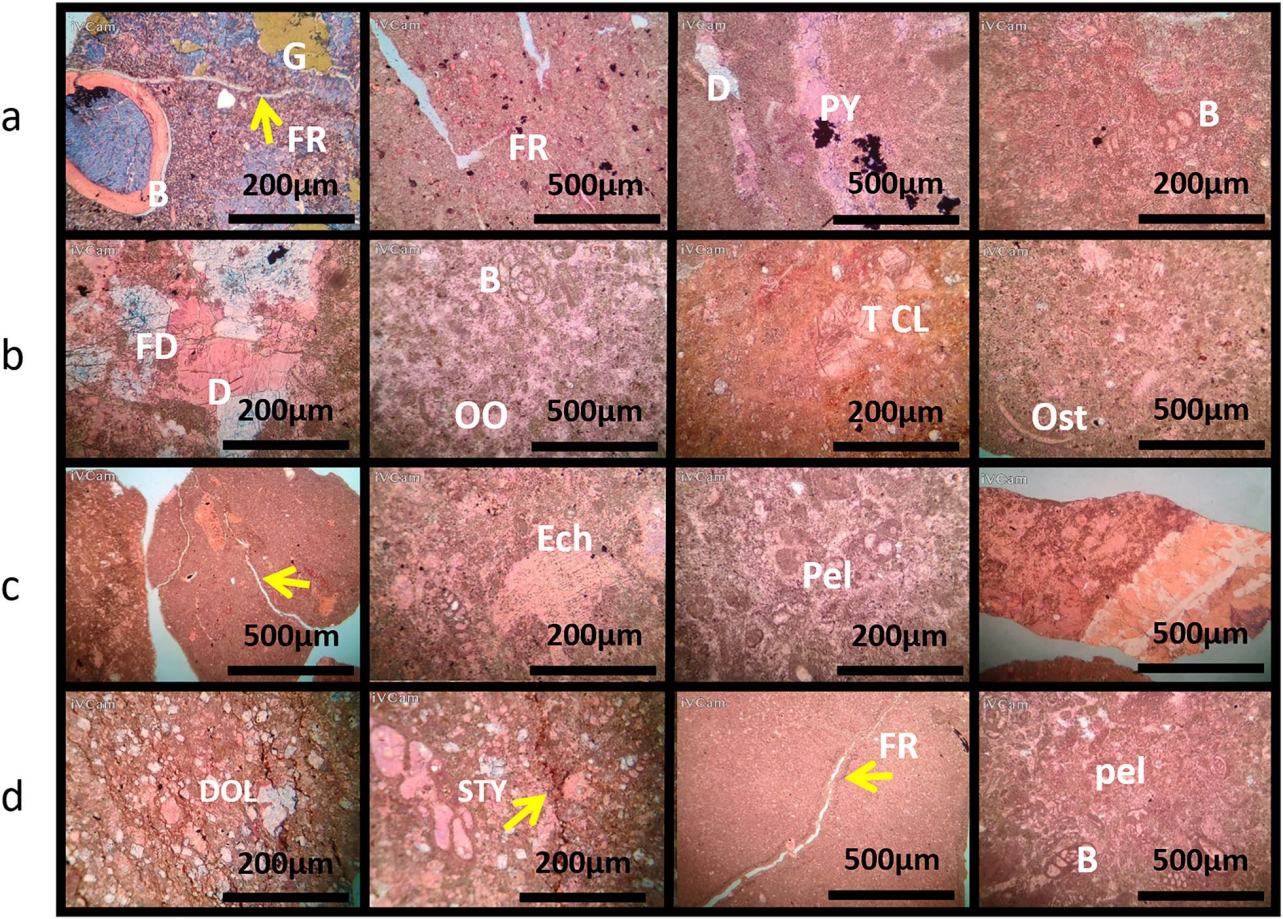
Petrographic descriptions and reservoir qualities of rock samples from different depths in the well. (**a**) Depth range 1671–74 MD showing MUD-WACKSTONE with a minor presence of benthic foraminifera, very minor terrigenous clays, and rare occurrences of various minerals. Reservoir quality is assessed as good. (**b**) Depth range 1674–77 MD displaying WACKSTONE with minor benthic foraminifera and terrigenous clays, and rare occurrences of other minerals, indicating moderate reservoir quality. (**c**) Depth range 1677–1680 MD exhibiting WACKSTONE with minor terrigenous clays, very minor dolomite and benthic foraminifera, and rare occurrences of other minerals, with a moderate reservoir quality. (**d**) Depth range 1680–83 MD showing WACKSTONE with minor terrigenous clays, pelloides, and benthic foraminifera, very minor dolomite and echinoids, and rare occurrences of pyrite and ferroan dolomite, indicating a moderate reservoir quality.

**Figure 6:**
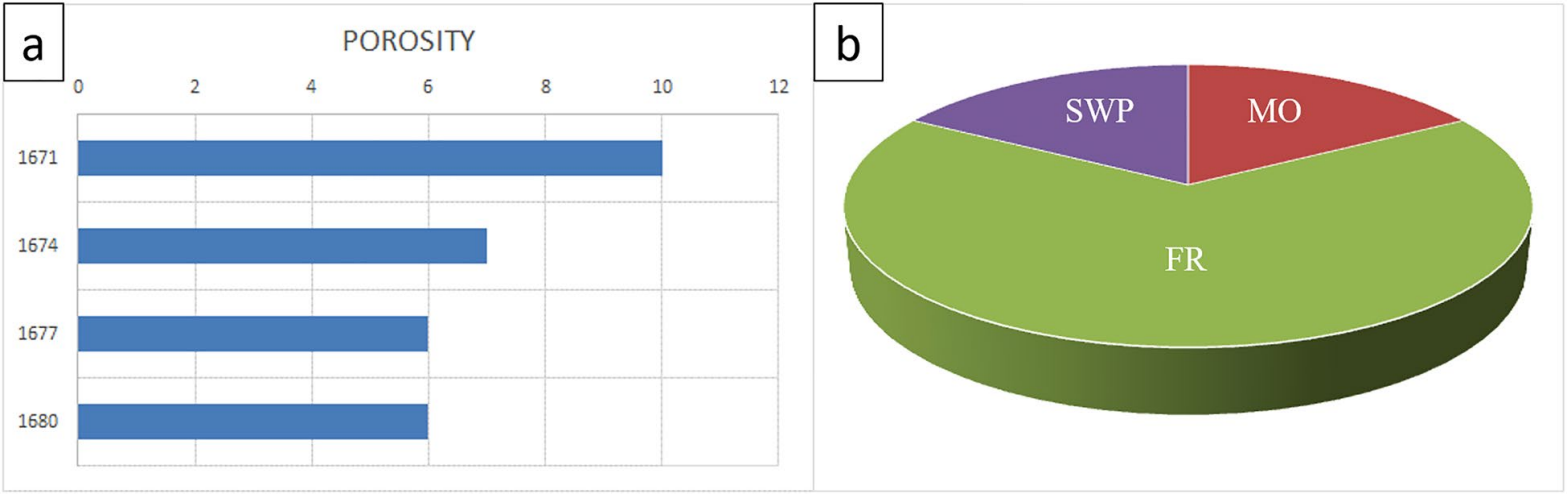
Well-01 analysis (**a**) Porosity Chart depicting the relationship between depth (Y-axis) and porosity percentage (X-axis), illustrating variations in subsurface porosity. (**b**) Pie chart illustrating the distribution of different types of porosities.

The sedimentary profile reveals a wackstone rock type dominated by terrigenous clays, pelloides, and benthic foraminifera. The rock exhibits a minor presence of dolomite and Echinoides. Although rare elements like pyrite, residual hydrocarbon, and ferroan dolomite are present in small quantities, the reservoir quality is assessed as moderate, suggesting potential hydrocarbon accumulation with some geological challenges. The petrographic examination highlights dynamic transitions from mud-wackstone to wackstone, providing crucial insights into the geological framework of the AR/D Member (Figs. 5 and 6).

### AR/D member petrography analysis in well-02

**Figure 7:**
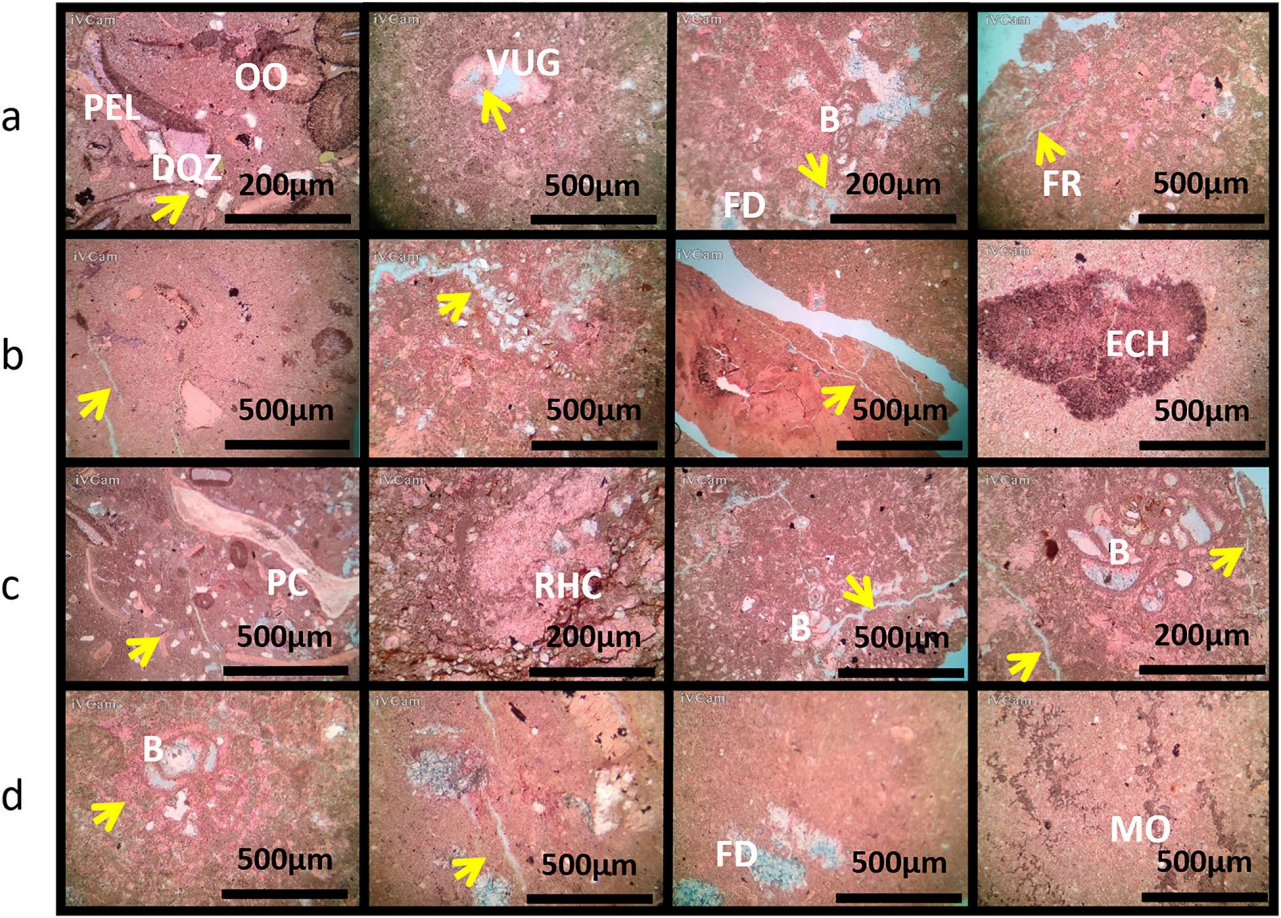
Petrographic thin sections from Well-02. (**a**) 1740–43 MD Plate Description: Minor of Ooids, Echinoides, benthic foraminifera, detrital quartz, terrigenous clays, and dolomite. Very minor of pelecypods, pyrite, and glucony. Rare of ferroan calcite and ferroan dolomite. Reservoir quality is good. Rock Name: PACKSTONE. (**b**) 1743–46 MD Plate Description: Minor of terrigenous clays. Very minor of Echinoides, pelecypods, and pyrite. Rare of ferroan calcite. Reservoir quality is moderate. Rock Name: WACKSTONE. (**c**) 1746–49 MD Plate Description: Minor of Ooids, pelecypods, Echinoides, benthic foraminifera, detrital quartz. Very minor residual hydrocarbon and pyrite. Rare of ferroan dolomite, glucony, and ostracods. Reservoir quality is good. Rock Name: PACKSTONE. (**d**) 1749–52 MD Plate Description: Minor of terrigenous clays, algae, and Ooids. Very minor of benthic foraminifera, dolomite, ferroan calcite, pyrite, detrital quartz, Echinoides, and pelecypods. Rare of ostracods. Reservoir quality is bad. Rock Name: WACKSTONE.

**Figure 8:**
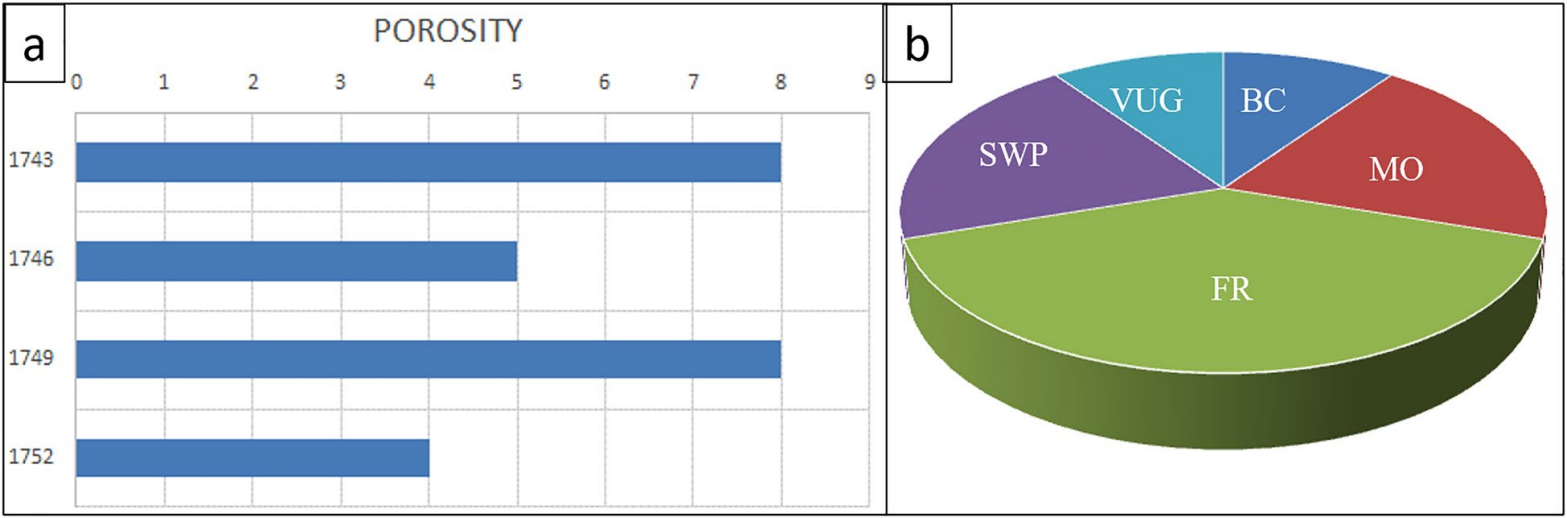
Well-02 analysis (**a**) Porosity Chart depicting the relationship between depth (Y-axis) and porosity percentage (X-axis), illustrating variations in subsurface porosity. (**b**) Pie chart illustrating the distribution of different types of porosities.

Figures 7 and 8 illustrate petrographic thin section analysis for Well-02, covering intervals between 1740 and 52 m. The observations reveal the complex composition and reservoir characteristics of the AR/D Member, showcasing a dynamic transition from mud-wackstone to wackstone. Various components, including echinoides, ostracods, detrital quartz, dolomite, ferroan calcite, pelecypodes, ferroan dolomite, pyrite, phosphatic fragments, and glaucony, influence the reservoir quality, categorized as good to moderate across different intervals.

#### The depth interval of 1740-43 meters

The sedimentary profile is characterized by a diverse composition, including major proportions of ooids, echinoides, benthic foraminifera, detrital quartz, terrigenous clays, and dolomite. Despite the varied composition, the reservoir quality is deemed good, promising favorable conditions for potential resource extraction.

#### The depth interval of 1743-46 meters

The sedimentary profile is dominated by terrigenous clays, forming a wackstone rock type. There are trace amounts of Echinoides, pelecypods, and pyrite, with rare occurrences of ferroan calcite. The reservoir quality is assessed as moderate, indicating a moderate potential for fluid storage and flow within this geological formation.

#### The depth interval of 1746-49 meters

The sedimentary profile is characterized by a packstone rock type, exhibiting a minor presence of ooids, pelecypods, Echinoides, and benthic foraminifera. Despite minimal occurrences of detrital quartz, residual hydrocarbon, and pyrite, the reservoir quality is deemed good, reflecting favorable conditions for potential resource extraction or further geological studies.

#### The depth interval of 1749-52 meters

The prevailing geological composition is encapsulated within a wackstone rock type. This sedimentary unit primarily consists of terrigenous clays, algae, and ooids, indicating a depositional environment influenced by a mixture of terrestrial and marine processes. Despite the diverse sedimentary components, the reservoir quality of the rock is deemed unfavorable, pointing towards suboptimal conditions for fluid flow and extraction.

### AR/D member petrography analysis in well-03

**Figure 9:**
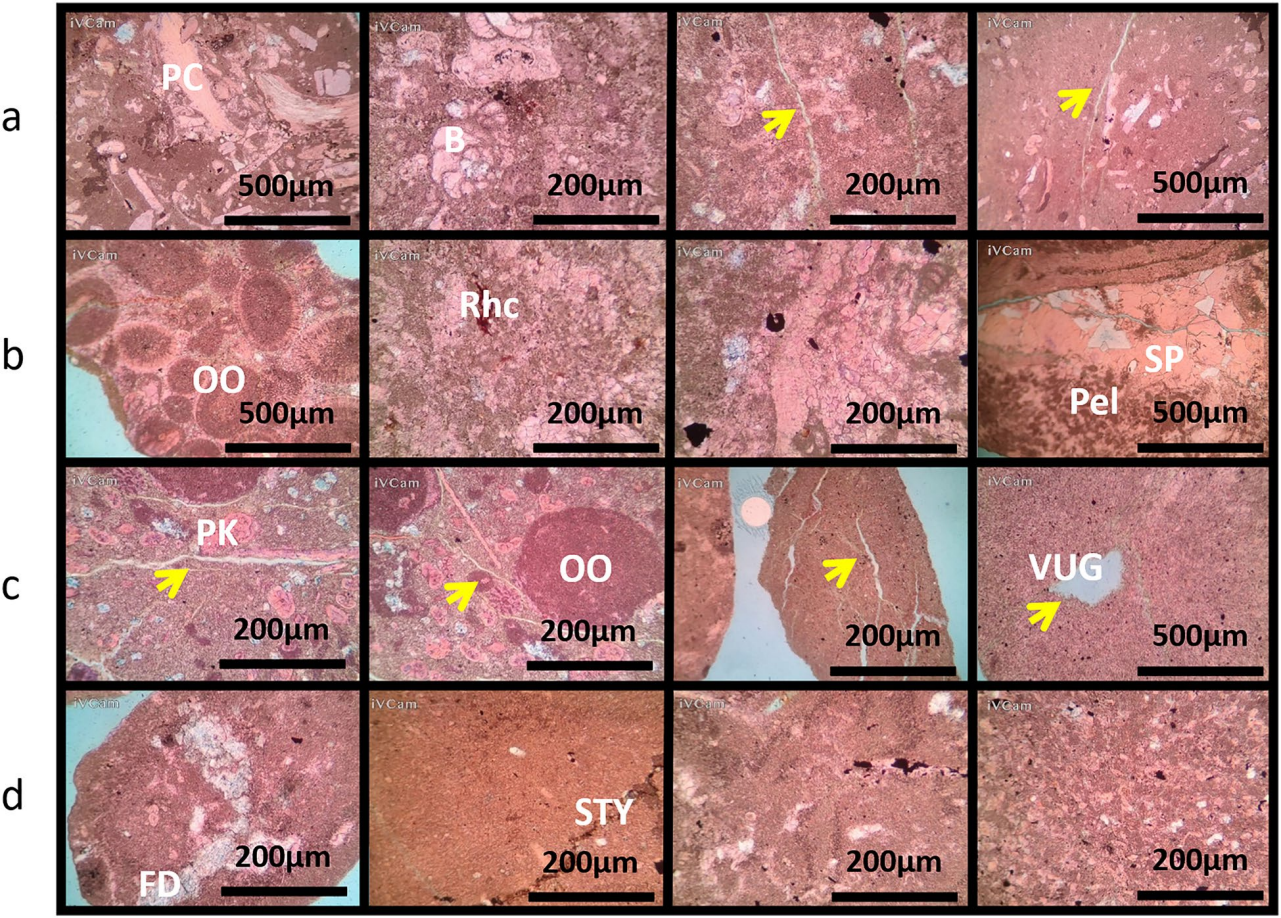
Petrographic analysis of Well-03. (**a**) 1662–1665 MD Plate Description: Common benthic foraminifera, minor of Ooids, Echinoides, terrigenous clays, pelecypods dolomite, and pyrite. Very minor of ostracods and ferroan calcite. Rare of ferroan dolomite. Reservoir quality is good. Rock Name: PACKSTONE. (**b**) 1665–1668 MD Plate Description: Common ooids, minor of benthic foraminifera, terrigenous clays, and chert. Very minor of pelecypods, detrital quartz, and pyrite. Rare of ferroan calcite and ostracods. Reservoir quality is bad. Rock Name: OOLITIC WACKSTONE. (**c**) 1668–1671 MD Plate Description: Common ooids, minor of benthic foraminifera, and terrigenous clays. Very minor of pelecypods, dolomite, and ferroan dolomite. Rare of ostracods and ferroan calcite. Reservoir quality is good. Rock Name: OLITIC PACKSTONE. (**d**) 1671–1674 MD Plate Description: Minor of terrigenous clays. Very minor of dolomite and ferroan dolomite. Rare of benthic foraminifera, Echinoides, ooids, glucony, and pyrite. Reservoir quality is bad. Rock Name: MUDSTONE.

**Figure 10:**
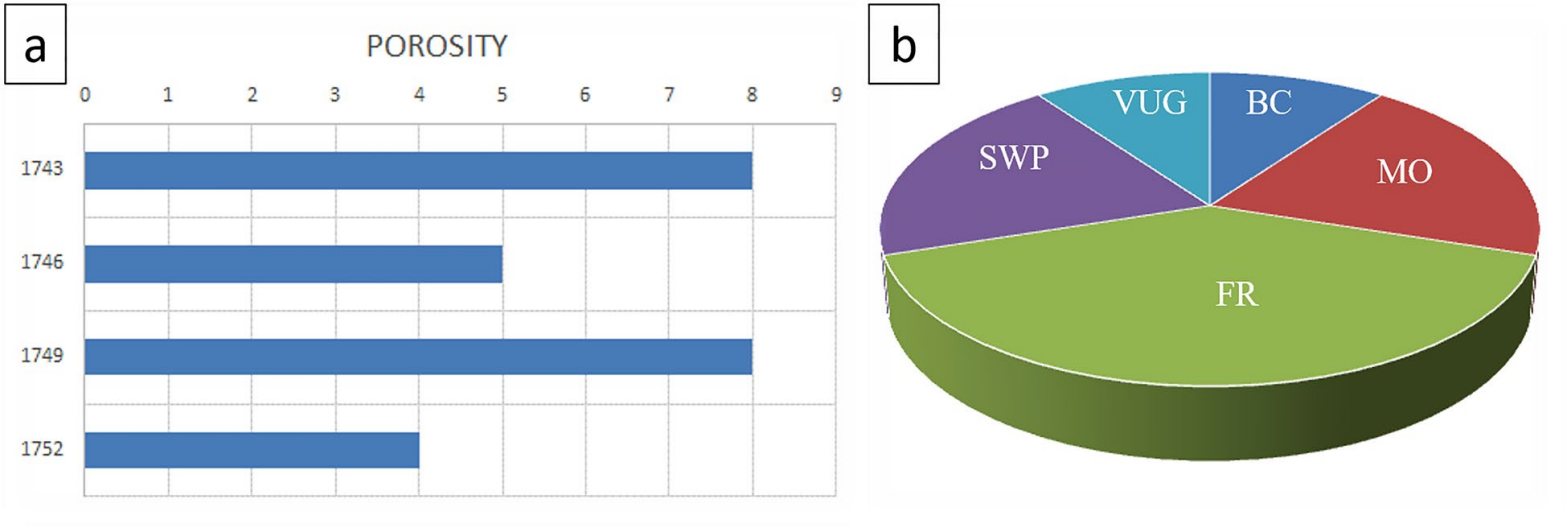
Well-03 analysis (**a**) Porosity Chart depicting the relationship between depth (Y-axis) and porosity percentage (X-axis), illustrating variations in subsurface porosity. (**b**) Pie chart illustrating the distribution of different types of porosities.

Figures 9 and 10 present the petrographic analysis of Well-03, showcasing transitions from packstone to oolitic-packstone and varied lithological compositions and their impact on reservoir quality. This emphasizes the importance of considering different facies in evaluating the hydrocarbon potential of the AR/D reservoir.

#### The depth interval of 1662-65 meters

The sedimentary profile reveals a distinctive packstone rock type dominated by common benthic foraminifera, with minor components of ooids, echinoides, terrigenous clays, pelecypods, dolomite, and pyrite. Ostracods and ferroan calcite are present in very minor quantities, while ferroan dolomite is exceptionally rare. The reservoir quality within this interval is assessed as good, indicating favorable conditions for fluid storage and migration.

#### The depth interval of 1665-68 meters

The geological composition is characterized by an oolithic wackstone, predominantly comprising common ooids, with minor quantities of benthic foraminifera, terrigenous clays, and chert. Despite the diverse nature of the sediment, the reservoir quality is described as poor, indicating limitations in the potential for hydrocarbon extraction.

#### The depth interval of 1668-71 meters

The rock formation is characterized as an oolitic packstone, revealing common ooids with minor occurrences of benthic foraminifera and terrigenous clays. Very minor proportions of pelecypods, dolomite, ferroan dolomite, rare ostracods, and ferroan calcite are noted. Despite the varied components, the reservoir quality of this oolitic packstone is deemed good.

#### The depth interval of 1671-74 meters

The geological composition is primarily represented by mudstone, consisting of terrigenous clays with minor occurrences of dolomite, ferroan dolomite, benthic foraminifera, Echinoides, ooids, glucony, and pyrite. Unfortunately, the reservoir quality of this geological formation is characterized as poor due to the prevalence of mudstone and limited occurrences of other minerals and microorganisms, making it challenging for hydrocarbon exploration and extraction.

### AR/D member petrography analysis in well-04

**Figure 11:**
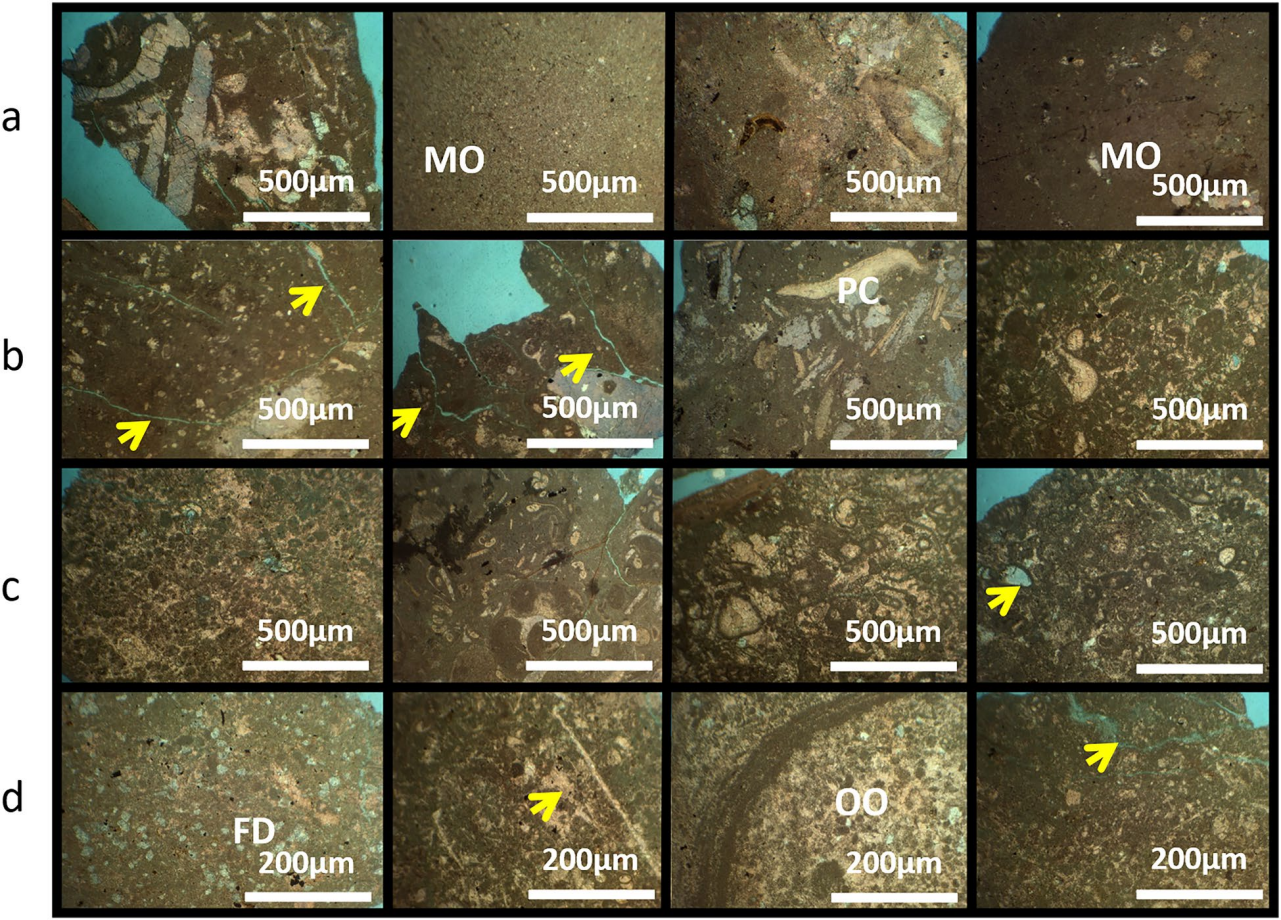
Petrographic analysis of the AR/D Member in Well-04. (**a**) 1761–1764 MD Plate Description: Common f terrigenous clays, Minor of Echinoides, Very minor of pyrite, Rare of dolomite. Reservoir quality is bad. Rock Name: MUD-WACKSTONE. (**b**) 1764–1767 MD Plate Description: Common peloids, Minor of Echinoides, Very minor of pelecypods and ostracods, Rare of pyrite. Reservoir quality is good. Rock Name: PELLOIDS WACK-PACKSTONE. (**c**) 1767–1770 MD Plate Description: Common peloids, Minor of Echinoides, Very minor of ostracods, Rare of residual hydrocarbon. Reservoir quality is bad. Rock Name: PACKSTONE. (**d**) 1770–1773 MD Plate Description: Common ooids, Minor of terrigenous clays, Very minor of pyrite. Reservoir quality is moderate. Rock Name: OOLITIC PACKSTONE.

**Figure 12:**
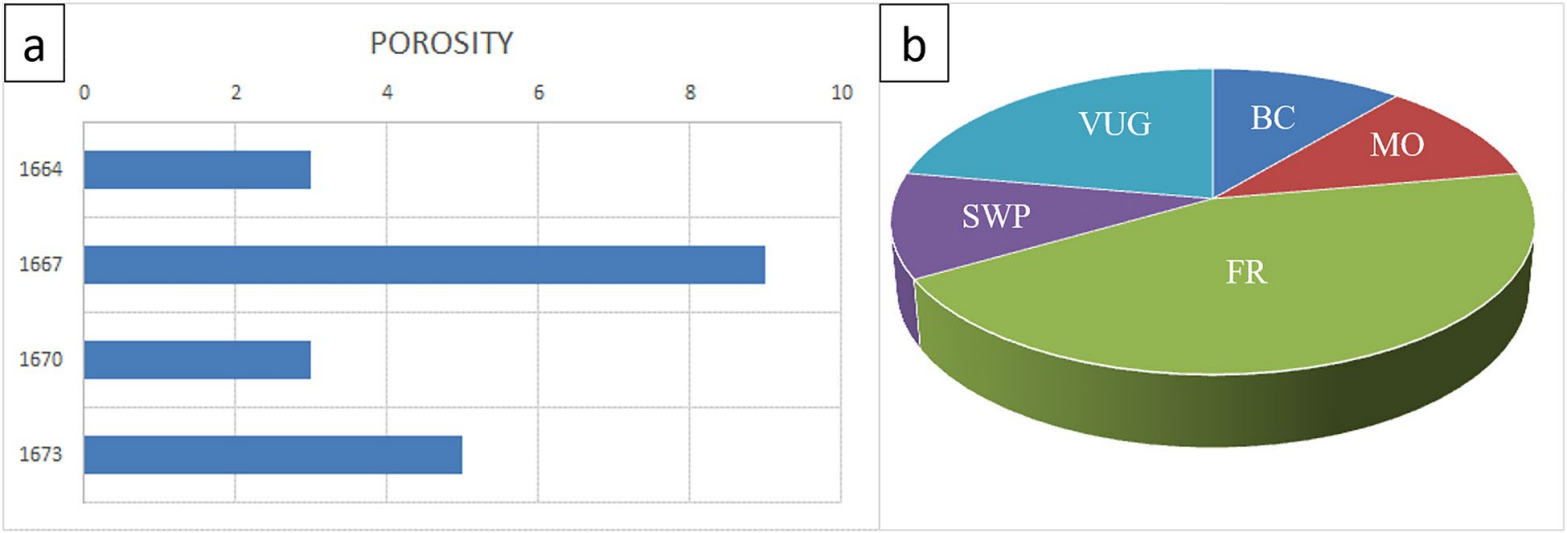
Well-04 analysis (**a**) Porosity Chart depicting the relationship between depth (Y-axis) and porosity percentage (X-axis), illustrating variations in subsurface porosity. (**b**) Pie chart illustrating the distribution of different types of porosities.

Petrographic thin sections and visual porosity analysis (Figs. 11 and 12) from Well-04 reveal dynamic transitions from mud-wackstone to oolitic-packstone.

#### The depth interval of 1761-64 meters

The plate description indicates the predominant presence of common fine terrigenous clays, with minor occurrences of Echinoides. There is a very minor presence of pyrite and rare occurrences of dolomite, suggesting poor reservoir quality. The rock type is identified as mud-wackstone, reflecting a lithological classification characterized by a matrix of mud-sized particles.

#### The depth interval of 1764-67 meters

The plate characterizes the rock as pelloids wack-packstone, with common peloids, minor occurrences of Echinoides, and a very minor presence of pelecypods and ostracods. Despite the sedimentary components, the reservoir quality is deemed good.

#### The depth interval of 1767-70 meters

The plate analysis reveals a composition characterized by common peloids, a minor presence of Echinoides, and very minor occurrences of ostracods. Rare residual hydrocarbons are observed, but the overall reservoir quality is described as poor. The rock type associated with this depth interval is identified as packstone.

#### The depth interval of 1770-73 meters

The rock prevalent in this depth range is identified as Oolitic Packstone, showcasing a composition dominated by common ooids. There are minor components of terrigenous clays, and very minor amounts of pyrite are noted. The reservoir quality within this interval is considered moderate, indicating the potential for fluid movement within the rock formation.

### Seismic interpretation analysis

**Figure 13:**
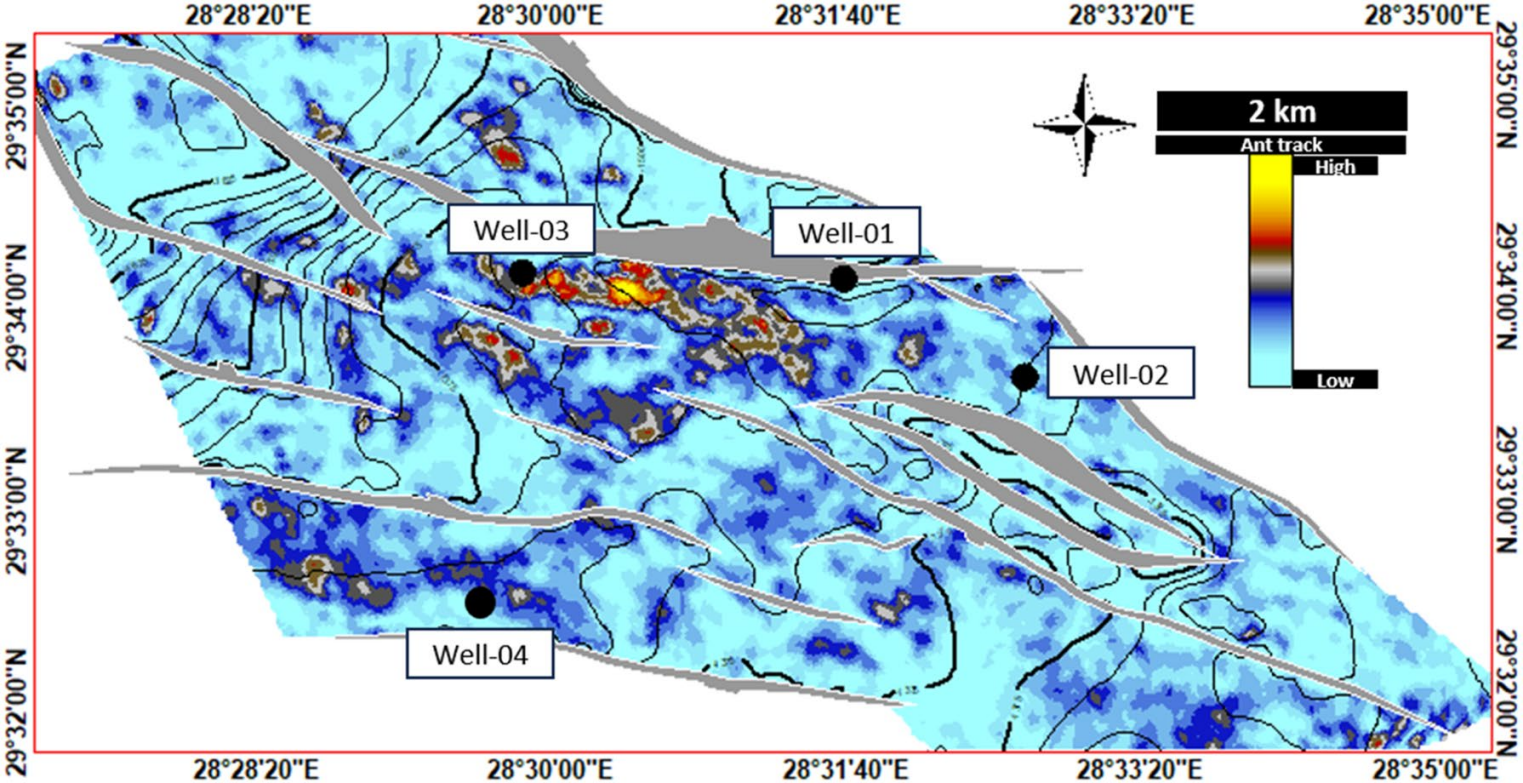
RMS Average Magnitude Amplitude Map of Upper Cretaceous AR/D Member.

**Figure 14:**
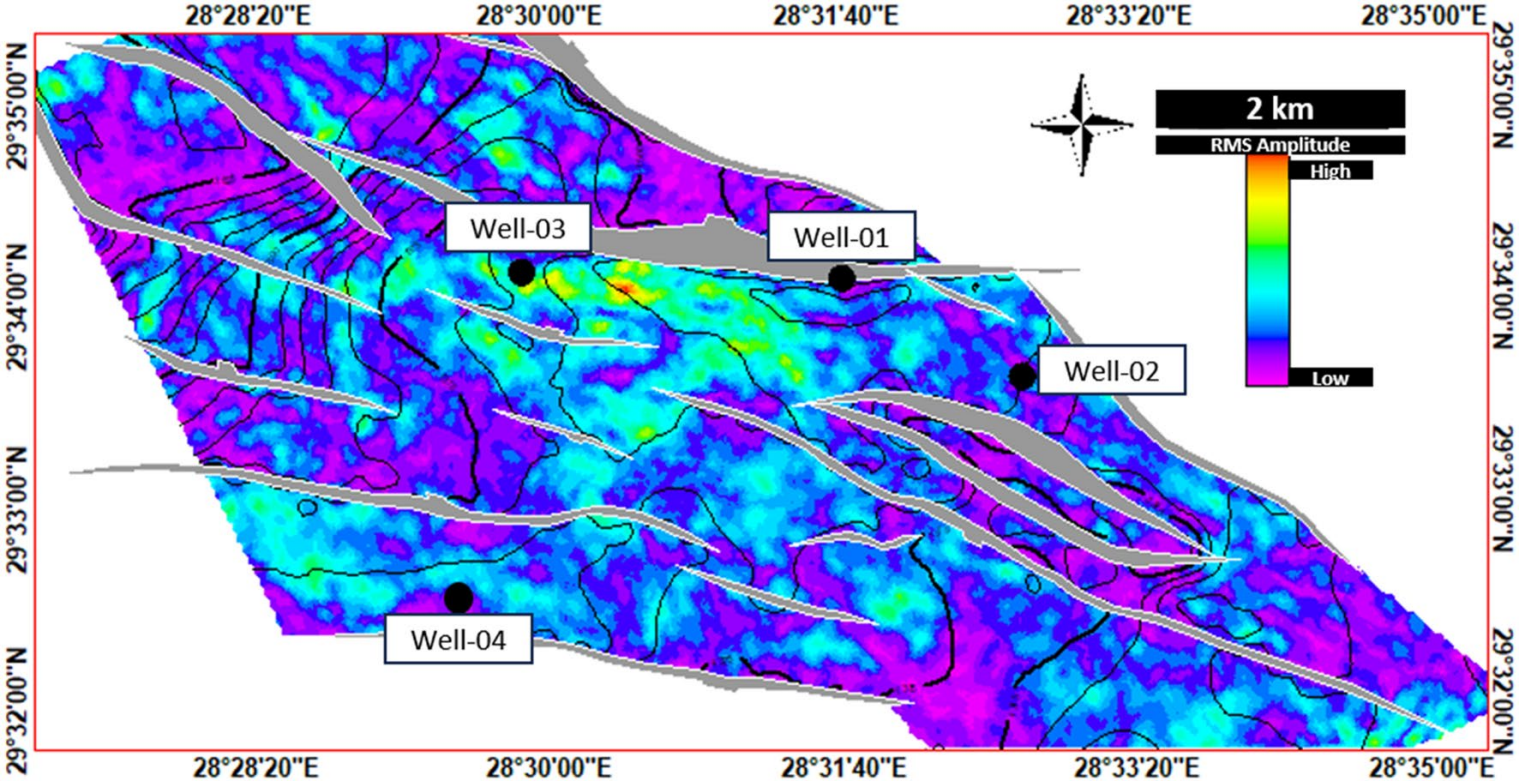
Ant track Extracted Value Map of Upper Cretaceous AR/D Member.

**Figure 15:**
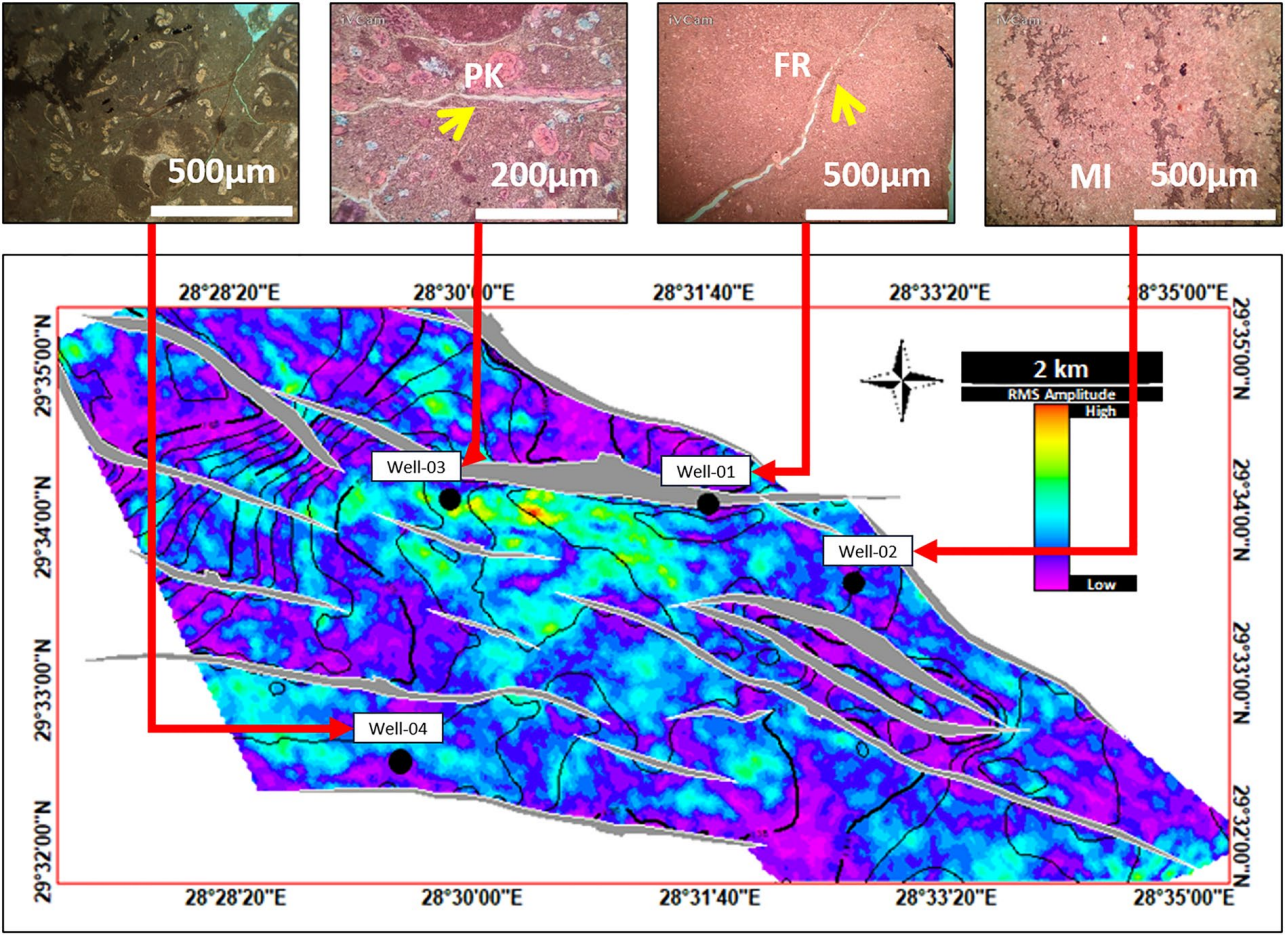
Integrated Petrography and seismic analysis of the AR/D carbonate reservoir through the utilization of the RMS seismic attribute surface depicting the relative facie distribution across the study area against the controlling structural elements, highlighting a distinct anomaly in its central region.

References^7–10^published a series of papers regarding the geological assessment/hydrocarbon potential of the Southern Abu Ghardig region, with a focus on the Upper Cretaceous Members. Through their geo-evaluation, they uncovered NE-SW folds resembling 3-way closures, enclosed by adjacent extensional faults. These closures, identified as effective traps for hydrocarbons, align with our study’s Seismic/subsurface mapping of the Upper Cretaceous carbonate members (AR/D). Our study extended the extensive analysis of the reservoir characteristics of the Upper Cretaceous carbonate AR/D member by integrating petrography and seismic analysis and used the Ant track (Fig. 13) and RMS (Figs. 14 and 15) seismic attribute surfaces on the AR/D carbonate reservoir showed relative facie distribution of the AR/D carbonate reservoir in the study area, with a clear anomaly in the central part of the study area.

In analyzing the structural level of AR/D concerning our wells, it’s evident that Well-03 stands out due to its relatively high-altitude structural position, drilled near a major fault, revealing distinct fracture sets that contribute to a notably high reservoir quality as depicted in the RMS amplitude and Ant track attributes maps illustrated in Figs. 13 and 14. These observations are matched with the petrography analysis performed. Well-01 exhibits conditions similar to Well-03, situated in the central highly fractured part of the study area where high amplitude/phenomenal anomaly is preserved. Conversely, Wells 02 and 04 are relatively positioned at structurally lower levels, and face challenges with overburden pressure and mechanical compaction, resulting in diminished facies quality^32^ for the reservoir (Fig. 15).

## Discussion

Expanding on Noureldin’s previous work in structural, stratigraphical, and petroleum system analysis^7–10^, this work extends the subsurface assessment to encompass a broader characterization of the Upper Cretaceous carbonate member AR/D. Drawing on accumulated expertise in this field, the aim is to further detail the mapping of AR/D Upper Cretaceous carbonate members, with a particular focus on enhancing characterization through petrographic and seismic analysis. This study is compared to^33^’s findings that focus on the seismic interpretation of the Abu Roash D Member, emphasizing the tectonic history and the relationship between fractures and normal faults.

## Conclusion

The study aimed to assess the composition and reservoir quality of the Upper Cretaceous member AR/D’s carbonate member in the Southern Abu Gharadig Basin within the Northern Western Desert of Egypt. The findings of this research follow:

- The study delves into the geological intricacies of the AR/D carbonate reservoir in the SWS oil field in the Southern Abu Gharadig Basin, Egypt.
- Integration of petrographic analysis, electrical well logs, and seismic data provides insights into composition, lithology, and the controlling structure of the target reservoir.
- Overcoming challenges in well placement and facies identification, the study establishes a foundation for further exploration in the region.
- Petrographic analysis reveals transitions from mud-wackstone to wackstone, packstone, and oolitic packstone, influencing reservoir quality.
- Diagenesis processes such as dolomitization and dissolution refine the understanding of the geological framework.
- Well analysis:
  - Well-01 exhibits mud-wackstone with various mineral components at 1671-74 meters MD, indicating good reservoir quality.
  - Well-02 shows diverse compositions at intervals 1740-43 meters MD and 1746-49 meters MD, with good reservoir quality.
  - Well-03 reveals a packstone rock type at 1662-65 meters MD with good reservoir quality.
  - Well-04 displays peloids Wack-Packstone and Oolitic Packstone at intervals 1764-67 meters MD and 1770-73 meters MD, respectively, both with good reservoir quality.
- Seismic interpretation highlights structural complexity, including an asymmetrical anticline intersected by normal faults. Seismic attributes like the Ant track and RMS amplitude aid in characterizing petrophysical properties and confirming hydrocarbon potential. Fractures within the AR/D carbonate, correlated with faults, act as structural traps for hydrocarbons.
- Insights gained from the study can extend to neighboring areas, enhancing hydrocarbon exploration potential.

## Data Availability

Data are available from the corresponding author upon reasonable request and with permission of (The Egyptian General Petroleum Cooperation).

## Abbreviations

G: glaucony
Py: pyrite
B: benthic
FD: ferroan dolomite
OO: ooids
D: dolomite
TCL: terrigenous clays
Ost: Ostracods
Ech: Echinoids
Pel: Peloids
Sty: Stylolites
PC: Pelecypods
Rhc: residual hydrocarbon
SP: sprite
PK: planktonic
Mi: micrite
FR: fracture porosity
MO: moldic porosity
SWP: secondary within particles porosity
BC: between crystals porosity
VUG: vuggy porosity
